# Psychological vulnerability and problematic psychotropic drug use among medical residents: exploring the relationship

**DOI:** 10.1192/j.eurpsy.2024.1458

**Published:** 2024-08-27

**Authors:** H. Ktari, S. Sellami, J. Ben Thabet, S. Omri, R. Feki, I. Gassara, N. Smaoui, L. Zouari, M. Maalej, M. Maalej Bouali, N. Charfi

**Affiliations:** Psychiatry C, University Hospital Hedi Chaker, Sfax, Tunisia

## Abstract

**Introduction:**

Psychological vulnerability and problematic psychotropic drug use among medical residents are critical and intricate areas of study in the field of healthcare and mental well-being. This topic looks into the potential links between the psychological vulnerabilities experienced by medical residents, which are frequently associated with the demanding nature of their profession, and their use of psychotropic drugs in a way that poses problems or risks. Exploring this relationship is critical for understanding the mental health challenges that medical residents face and developing effective strategies to support their psychological well-being.

**Objectives:**

to identify the psychological factors linked to problematic psychotropic drug use in medical residents.

**Methods:**

We conducted a cross-sectional descriptive and analytical study among Tunisian medical residents between August and September 2022. We used a self-administered questionnaire with a data collection form, the DAST-10 (Drug Abuse Screening Test) scale, and the DASS-21 (Depression, Anxiety, and Stress Scale) in an online survey. Data was analyzed using the 20th version of the SPSS software.

**Results:**

The sample consisted of 80 medical residents. Among them, 23.8% (n=19) had reported a previous use of psychotropic drugs, and 15% (n=12) a misuse (without a prescription and/or without following the prescription). The DAST-10 revealed that 6 residents (31.6%) had problematic use of psychotropic drugs.

A high level of stress on the DASS-21 scale was associated with a problematic use (p=0.01) and a misuse (p=0.01) of psychotropic drugs. Furthermore, residents with high stress levels were more likely to demonstrate problematic use of psychotropic drugs (p=0.004). Such problematic use was correlated with personal history of anxiety disorders (p=0.01).

Furthermore, residents with problematic psychotropic drug use had higher anxiety and depression scores on the DASS-21 scale (p>0.05).

**Conclusions:**

Our findings revealed a concerning prevalence of psychotropic drug use among medical residents and an association with high stress levels. This result emphasizes the need for targeted interventions to support young doctors’ mental health.

**Disclosure of Interest:**

None Declared

